# Intubation Trends and Survival in Pediatric In-Hospital Cardiac Arrest

**DOI:** 10.1001/jamanetworkopen.2025.44365

**Published:** 2025-11-20

**Authors:** Lindsay N. Shepard, Ron W. Reeder, Jesse Hsu, Garrett Keim, Robert A. Berg, Robert M. Sutton, Alexis A. Topjian, Nadir Yehya, Ryan W. Morgan

**Affiliations:** 1 Department of Anesthesiology and Critical Care Medicine, Children’s Hospital of Philadelphia, Perelman School of Medicine at the University of Pennsylvania, Philadelphia; 2 Department of Pediatrics, University of Utah, Salt Lake City; 3 Department of Biostatistics and Epidemiology, Perelman School of Medicine at the University of Pennsylvania, Philadelphia

## Abstract

**Importance:**

The optimal airway management during pediatric in-hospital cardiac arrest (IHCA) is unknown.

**Objective:**

To evaluate intubation trends during pediatric IHCA between 2000 and 2022, and determine the association of intra-arrest intubation with survival in a recent cohort of patients (2017-2022).

**Design, Setting, and Participants:**

This retrospective cohort study (analysis performed between June 2023 and October 2024) used data from the multicenter American Heart Association Get With The Guidelines–Resuscitation registry. Pediatric patients (<18 years) with IHCA from 2000 through 2022 were included.

**Exposure:**

Intra-arrest endotracheal intubation.

**Main Outcomes and Measures:**

The primary outcome was survival to hospital discharge. Intra-arrest intubation trends were assessed using nonparametric test for trend. A time-dependent propensity matched analysis assessed the association between intra-arrest intubation and hospital survival from 2017 through 2022. Each minute, patients intubated were matched with patients at risk of intubation using a propensity score, with forced matching on stratification variables and replacement of controls. Mixed-effects logistic regression assessed the association with survival outcomes, with subgroup analysis by age and illness category.

**Results:**

The cohort included 3262 pediatric patients with IHCA (median age, 12.0 [IQR, 3.0-83.8] months; 1775 [54.4%] male) with no advanced airway at CPR onset. Return of spontaneous circulation was attained in 2413 patients (74.0%), and 1748 (53.6%) survived to hospital discharge. The intubation rate decreased over time (33 of 39 [84.6%] in 2000 to 112 of 168 [66.7%] in 2022; *P* < .001). In the 2017-2022 cohort, intubation vs nonintubation in each minute of CPR was associated with decreased discharge survival odds in unadjusted analysis (odds ratio [OR], 0.18; 95% CI, 0.14-0.24; *P* < .001) but not after matching (adjusted OR, 1.18; 95% CI, 0.90-1.53; *P* = .23). In children aged 8 years or older, after matching, intubation compared with nonintubation in each minute was associated with increased odds of discharge survival (adjusted OR, 1.91; 95% CI, 1.09-3.33; *P* = .02).

**Conclusions and Relevance:**

In this cohort study of pediatric patients with IHCA between 2017 and 2022 without an advanced airway at the start of CPR, no association was identified between intra-arrest tracheal intubation and hospital survival after time-dependent propensity score matching. In subgroup analysis, intra-arrest intubation in children 8 years or older was associated with higher survival odds. These findings may have important clinical implications for clinicians caring for children with IHCA and warrant further investigation into the physiologic and practical mechanisms of this association.

## Key Points

**Question:**

Should pediatric patients with in-hospital cardiac arrest (IHCA) be intubated during cardiopulmonary resuscitation?

**Findings:**

In this cohort study of 3262 children who experienced IHCA, rates of intra-arrest intubation decreased across the study period (2000-2022). Using time-dependent propensity-score matched analysis, there was no association between intra-arrest intubation and survival to hospital discharge in a recent (2017-2022) cohort of patients; however, intubation was associated with increased odds of survival to discharge in children aged 8 years or older.

**Meaning:**

Although there was no association between intra-arrest intubation and hospital survival overall, this study’s findings suggest that there are subpopulations of children who may benefit from intra-arrest intubation.

## Teaser

This cohort study examines intubation trends during in-hospital cardiac arrest among children in the US and evaluates the association of intra-arrest intubation with survival.

## Introduction

In-hospital cardiac arrest (IHCA) occurs in at least 15 000 children annually in the United States, mostly in children with respiratory failure.^1,2,3^ However, the optimal airway management strategy during pediatric IHCA is not known.^4^ Current American Heart Association pediatric life support guidelines state there is insufficient data to support a recommendation for or against advanced airway placement during IHCA, but there may be specific circumstances or populations in which early advanced airway interventions are beneficial.^4^

Although there is an absence of explicit guidance for intubation during IHCA, historically it has been prioritized during cardiopulmonary resuscitation (CPR).^5,6,7,8^ Despite it being common practice, the only published study of intra-arrest intubation in pediatric IHCA found an association between intra-arrest intubation and decreased survival to hospital discharge.^8^ It is plausible that the potential adverse effects of intra-arrest intubation during pediatric IHCA may have been mitigated by the impact of the prior work as well as an increased emphasis on high-quality pediatric CPR, including minimizing interruptions to chest compressions,^9,10^ improvements in hemodynamics during pediatric CPR,^11,12^ prompt epinephrine administration,^13,14^ and improvements in outcomes.^2,11,12,15^ We speculate that intra-arrest intubation may allow for superior oxygenation and ventilation and thus improve outcomes for some pediatric patients with IHCA. Key knowledge gaps remain about intubation during pediatric IHCA, including whether intra-arrest intubation practices have changed over time, whether the association between intra-arrest intubation and decreased survival is still evident, and whether there are associations between intra-arrest intubation and survival outcomes in specific pediatric subpopulations.

The objectives of this study were to evaluate trends in endotracheal intubation rates during pediatric IHCA between 2000 and 2022 and to determine the association of intra-arrest intubation and survival outcomes in a recent cohort of patients (2017-2022). We hypothesized that intubation rates would decrease over the study period and that intra-arrest intubation would be associated with increased odds of survival to hospital discharge.

## Methods

### Study Design

We performed a retrospective, multicenter, observational cohort study of children who received CPR for IHCA. The study protocol was determined not to be human participant research by the Children’s Hospital of Philadelphia Institutional Review Board; therefore, informed consent was not required. All hospitals in the American Heart Association’s Get With The Guidelines–Resuscitation (GWTG-R) database are required to comply with local regulatory guidelines. The reporting of this study follows the Strengthening the Reporting of Observational Studies in Epidemiology (STROBE) reporting guideline.

### Data Source

Data were analyzed from the GWTG-R database, a large, prospective, multicenter quality improvement registry of IHCA events in the US. Details of the GWTG-R registry design, Utstein-style data collection for uniform reporting of cardiac arrest events, and quality assurance have previously been described.^15,16,17^

### Study Population

We included children younger than 18 years with an index IHCA event that lasted at least 1 minute and without an invasive airway at the time of CPR initiation in the GWTG-R database from January 1, 2000, to December 31, 2022. Patients were excluded if (1) an advance directive limited intubation or CPR; (2) the arrest location was the delivery room, newborn nursery, or neonatal intensive care unit, age was less than 60 minutes, or the patient was categorized as “newly born” in the registry, given established differences in perinatal transitional vs pediatric physiology and the approach to airway management during CPR in these populations^18,19^; (3) the arrest was in the operating room, cardiac catheterization laboratory, or other diagnostic or intervention area; (4) the illness category was trauma, obstetric, or missing; (5) the patient was a visitor or employee; (6) an advanced airway was placed in the first minute (ie, minute zero) of CPR as intubation may have occurred prior to CPR; or (7) the presence of an invasive airway before the arrest was unknown, data were missing or conflicting regarding whether an advanced airway was placed during the CPR event or the timing of intubation, the event duration was more than 180 minutes, or the event or hospital survival outcome was missing or conflicting.

### Primary and Secondary Outcomes

When examining trends in intra-arrest intubation over time, the primary outcome was intra-arrest intubation, and the secondary outcome was time from start of CPR to intubation. When examining the association of intra-arrest intubation with outcomes, the primary outcome was survival to hospital discharge, and the secondary outcome was sustained return of spontaneous circulation (ROSC). ROSC was defined as not requiring CPR or extracorporeal membrane oxygenation for at least 20 minutes.^16^ Survival with favorable neurologic outcome was defined as a pediatric cerebral performance category (PCPC) of 1 (no disability) or 2 (mild disability), or no change from baseline at the time of discharge.^20^ Given anticipated missingness in the neurologic outcome variable (PCPC score among survivors)^8,21^ and that this missingness was not at random (ie, only surviving patients can have missing neurologic outcome), the analysis of the association of intra-arrest intubation and favorable neurologic outcome was treated as a sensitivity analysis.

### Statistical Analysis

We anticipated a similar absolute difference in survival (36% vs 41%, equivalent to a relative risk of 0.9) as reported by Andersen et al.^8^ Thus, we anticipated needing 2972 patients (1486 matched pairs) to achieve 80% power with a 2-sided α of .05.

The study population was characterized using descriptive statistics. Categorical variables are presented as counts and frequencies, and continuous variables as medians with IQRs. Categorical variables were compared using Fisher exact test and continuous variables using Wilcoxon rank sum test. Because supraglottic airway (SGA) placement was expected to be exceedingly rare, it was analyzed together with endotracheal intubation.

Full details of the statistical analysis are provided in the eMethods in Supplement 1. Briefly, annual trends for advanced airway placement and time to intubation were compared using a nonparametric trend test. To evaluate the association between intra-arrest intubation and survival to hospital discharge, a time-dependent propensity score–matched analysis was performed in the recent 2017-2022 cohort, in a historic cohort (2000-2016), and in the full cohort (2000-2022). Patients intubated in each minute were matched with patients at risk of intubation (ie, still receiving CPR and not yet intubated) in the same minute, based on a propensity score. Matching was performed with forced matching on 2 a priori–identified variables (age and illness categories) to allow for subgroup analyses, with replacement of controls. After matching, a mixed effects logistic regression model assessed the relationship between intra-arrest intubation and survival to hospital discharge, with weighting to account for the number of times a patient was included as a control, fixed effects to control for treatment at a high-volume center, and random effects to account for the matched data and clustering by hospital. Analyses were performed using Stata, 17/18 BE (StataCorp LLC) between June 2023 and October 2024.

## Results

**Figure 1.**
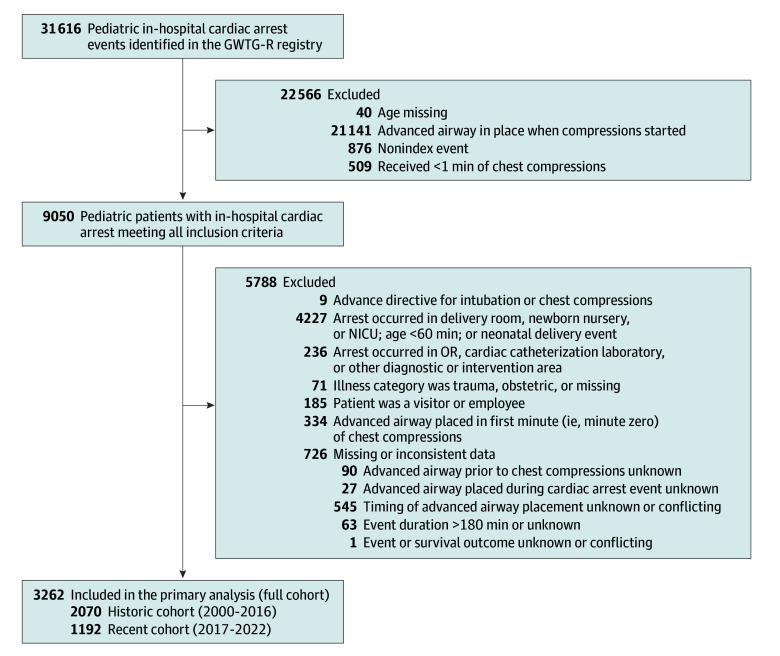
Patient Selection Flow Diagram Patient selection flow diagram indicating the number of pediatric in-hospital cardiac arrest events identified in the Get With The Guidelines—Resuscitation (GWTG-R) registry assessed for eligibility and included in the final cohort for analysis.

We identified 3262 children (median age of 12.0 [IQR, 3.0-83.8] months; 1487 [45.6%] female and 1775 [54.4%] male), in the GWTG-R database with IHCA without an advanced airway at the onset of CPR (Figure 1). Characteristics of the full study cohort are shown in Table 1 (recent and historical cohorts are in eTable 1 in Supplement 1). The median event duration was 16 (IQR, 5-37) minutes, the event outcome was ROSC in 2226 (68.2%), and 1748 (53.6%) survived to hospital discharge. The neurologic outcome variable was missing in 589 patients (ie, 18.1% overall and 33.7% of those who survived to hospital discharge).

**Table 1.** Characteristics of the Unmatched Study Population

Characteristic	Full cohort (2000-2022), No. (%)		
	Overall (N = 3262)	Intubated (n = 2164)	Not intubated (n = 1098)
Patient characteristics			
Age, median (IQR), mo	12.0 (3.0-83.8)	13.0 (4.0-95.8)	9.0 (3.0-59.9)
Age group			
<1 mo	180 (5.5)	118 (5.5)	62 (5.6)
1 mo to <1 y	1328 (40.7)	813 (37.6)	515 (46.9)
1 to <8 y	946 (29.0)	658 (30.4)	288 (26.2)
≥8 y	808 (24.8)	575 (26.6)	233 (21.2)
Sex			
Female	1487 (45.6)	982 (45.4)	505 (46.0)
Male	1775 (54.4)	1182 (54.6)	593 (54.0)
Illness category			
Cardiac	1303 (39.9)	913 (42.2)	390 (35.5)
Noncardiac	1959 (60.1)	1251 (57.8)	708 (64.5)
Preexisting conditions, No. (IQR)	1.5 (1-2)	1 (1-3)	2 (1-2)
None	394 (12.6)	266 (13.0)	128 (11.9)
Respiratory disorder^a^	1598 (51.0)	1033 (50.3)	565 (52.4)
Cardiac disorder^b^	1107 (35.3)	737 (35.9)	370 (34.3)
Neurologic disorder^c^	687 (21.9)	449 (21.9)	238 (22.1)
Shock	516 (16.5)	364 (17.7)	152 (14.1)
Congenital disorder^d^	482 (15.4)	258 (12.6)	224 (20.8)
Endocrine, metabolic, or electrolyte disorder^e^	463 (14.8)	318 (15.5)	145 (13.4)
Sepsis	302 (9.6)	213 (10.4)	89 (8.2)
Kidney disorder^f^	247 (7.9)	175 (8.5)	72 (6.7)
Oncologic disorder^g^	186 (5.9)	129 (6.3)	57 (5.3)
Hepatic disorder^h^	120 (3.8)	89 (4.3)	31 (2.9)
Missing preexisting conditions	129 (4.0)	110 (5.1)	19 (1.7)
Event characteristics			
Initial pulse status			
Pulseless	1765 (54.1)	1261 (58.3)	504 (45.9)
Pulse (poor perfusion)	1497 (45.9)	903 (41.7)	594 (54.1)
Initial rhythm			
Shockable	191 (5.9)	127 (5.9)	64 (5.8)
Nonshockable	2733 (83.8)	1838 (84.9)	895 (81.5)
Unknown	338 (10.4)	199 (9.2)	139 (12.7)
Event witnessed	2908 (89.1)	1908 (88.2)	1000 (91.1)
Location and time characteristics			
Event location			
Emergency department	640 (19.6)	522 (24.1)	118 (10.7)
Intensive care unit	1497 (45.9)	944 (43.6)	553 (50.4)
Floor (without telemetry)	508 (15.6)	288 (13.3)	220 (20.0)
Floor (with telemetry)	147 (4.5)	82 (3.8)	65 (5.9)
Other	470 (14.4)	328 (15.2)	142 (12.9)
Weekday daytime (Monday through Friday, 7 am to 11 pm)	1736 (53.2)	1160 (53.6)	576 (52.5)
Year			
2000	39 (1.2)	33 (1.5)	6 (0.5)
2001	55 (1.7)	46 (2.1)	9 (0.8)
2002	68 (2.1)	59 (2.7)	9 (0.8)
2003	88 (2.7)	66 (3.0)	22 (2.0)
2004	112 (3.4)	97 (4.5)	15 (1.4)
2005	126 (3.9)	87 (4.0)	39 (3.6)
2006	121 (3.7)	89 (4.1)	32 (2.9)
2007	135 (4.1)	101 (4.7)	34 (3.1)
2008	172 (5.3)	127 (5.9)	45 (4.1)
2009	161 (4.9)	125 (5.8)	36 (3.3)
2010	137 (4.2)	94 (4.3)	43 (3.9)
2011	111 (3.4)	75 (3.5)	36 (3.3)
2012	124 (3.8)	84 (3.9)	40 (3.6)
2013	123 (3.8)	82 (3.8)	41 (3.7)
2014	154 (4.7)	90 (4.2)	64 (5.8)
2015	168 (5.2)	101 (4.7)	67 (6.1)
2016	176 (5.4)	90 (4.2)	86 (7.8)
2017	207 (6.3)	116 (5.4)	91 (8.3)
2018	222 (6.8)	117 (5.4)	105 (9.6)
2019	211 (6.5)	135 (6.2)	76 (6.9)
2020	199 (6.1)	120 (5.5)	79 (7.2)
2021	185 (5.7)	118 (5.5)	67 (6.1)
2022	168 (5.2)	112 (5.2)	56 (5.1)
Hospital teaching status			
Major	1711 (52.7)	1070 (49.7)	641 (58.8)
Minor	1042 (32.1)	718 (33.3)	324 (29.7)
Nonteaching	76 (2.3)	66 (3.1)	10 (0.9)
Missing	416 (12.8)	301 (14.0)	115 (10.6)
Pediatric-only hospital	2455 (75.7)	1598 (74.2)	857 (78.6)
Event outcome			
Event duration, min (IQR)	16 (5-37)	28 (13-47)	3 (2-7)
Immediate outcome			
ROSC	2226 (68.2)	1215 (56.1)	1011 (92.1)
ROC via ECPR	230 (7.1)	223 (10.3)	7 (0.6)
Died	806 (24.7)	726 (33.5)	80 (7.3)
Survival to hospital discharge	1748 (53.6)	874 (40.4)	874 (79.6)

Abbreviations: ROC, return of circulation; ROSC, sustained return of spontaneous circulation; ECPR, extracorporeal cardiopulmonary resuscitation.

^a^ Pre-event respiratory insufficiency or pneumonia.

^b^ Pre-event cyanotic or acyanotic cardiac malformation, heart failure, or myocardial infarction.

^c^ Pre-event acute stroke or nonstroke neurologic event or baseline depression in neurologic function.

^d^ Noncardiac congenital malformation.

^e^ Pre-event metabolic or electrolyte abnormality or diabetes.

^f^ Pre-event kidney insufficiency.

^g^ Pre-event metastatic or hematologic malignancy.

^h^ Pre-event hepatic insufficiency.

### Trends in Intra-Arrest Advanced Airway Placement

**Figure 2.**
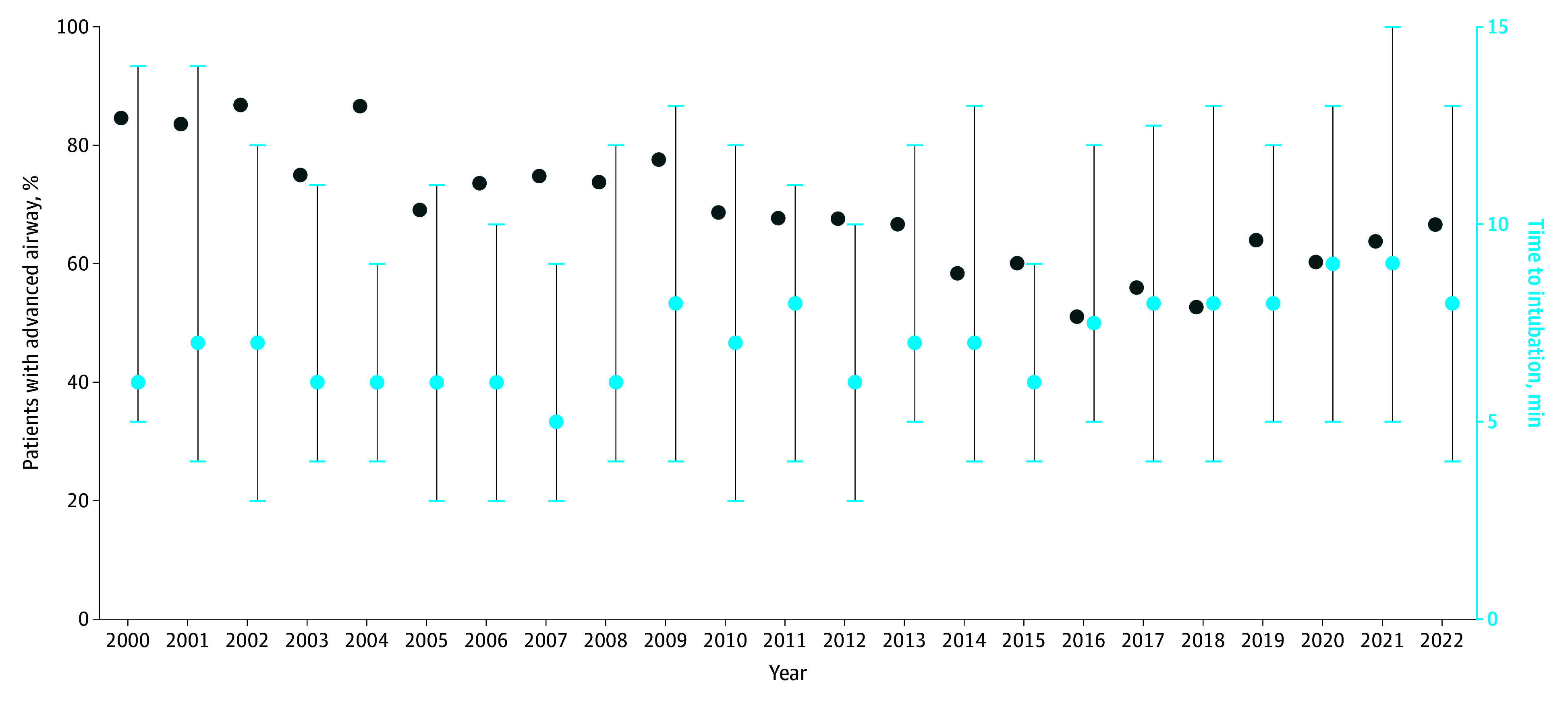
Trends in Intra-Arrest Advanced Airway Placement Trends in intra-arrest advanced airway placement by year, showing that the percentage of patients with intra-arrest airway placement (light blue circles, left y-axis) decreased by year (nonparametric test for trend, *P* < .001). The dark blue circles show the median time to intubation by year (right y-axis, with the whiskers representing the IQR), which increased over the study period (nonparametric test for trend, *P* < .001).

Overall, 2164 patients (66.3%) had an intra-arrest advanced airway placed, with 2139 (65.6%) receiving an endotracheal tube (ETT) only, 6 (0.18%) receiving an SGA only, and 10 (0.31%) receiving both an SGA and ETT. The median time to advanced airway was 7 (IQR, 4-12) minutes. In the recent cohort (2017-2022), 718 of 1192 (60.2%) received an intra-arrest advanced airway, and median time to advanced airway was 8 (IQR, 5-13) minutes. In the historical cohort (2000-2016), 1446 of 2070 (69.9%) received an intra-arrest advanced airway, and median time to advanced airway was 7 (IQR, 4-11) minutes. The proportion of patients with intra-arrest advanced airway placement decreased over time (from 33 of 39 [84.6%] in 2000 to 112 of 168 [66.7%] in 2022; nonparametric test for trend *P* < .001), and median time to advanced airway placement increased over time (from 6 [IQR, 5-14] minutes in 2000 to 8 [IQR, 4-13] minutes in 2022; nonparametric test for trend *P* < .001) (Figure 2). A post hoc evaluation of the trends in the historic (2000-2016) and recent (2017-2022) cohorts was performed after visual inspection of the shape of the graph. The proportion of patients with intra-arrest advanced airway placement decreased from 2000 to 2016 (from 33 of 39 [84.6%] to 90 of 176 [51.1%]; *P* < .001) and increased from 2017 to 2022 (116 of 207 [56.0%] in 2017 to 112 of 168 [66.7%] in 2022; *P* = .005).

Trends were similar in the sensitivity analysis of the less restrictive cohort (ie, including patients with missing or inconsistent data) (nonparametric test for trend, *P* < .001 for intra-arrest advanced airway placement and median time to airway placement). Trends in intra-arrest advanced airway placement in the primary cohort compared to the less restrictive cohort are shown in eFigure 1 in Supplement 1.

### Survival Analyses

#### Recent Cohort (2017-2022)

**Figure 3.**
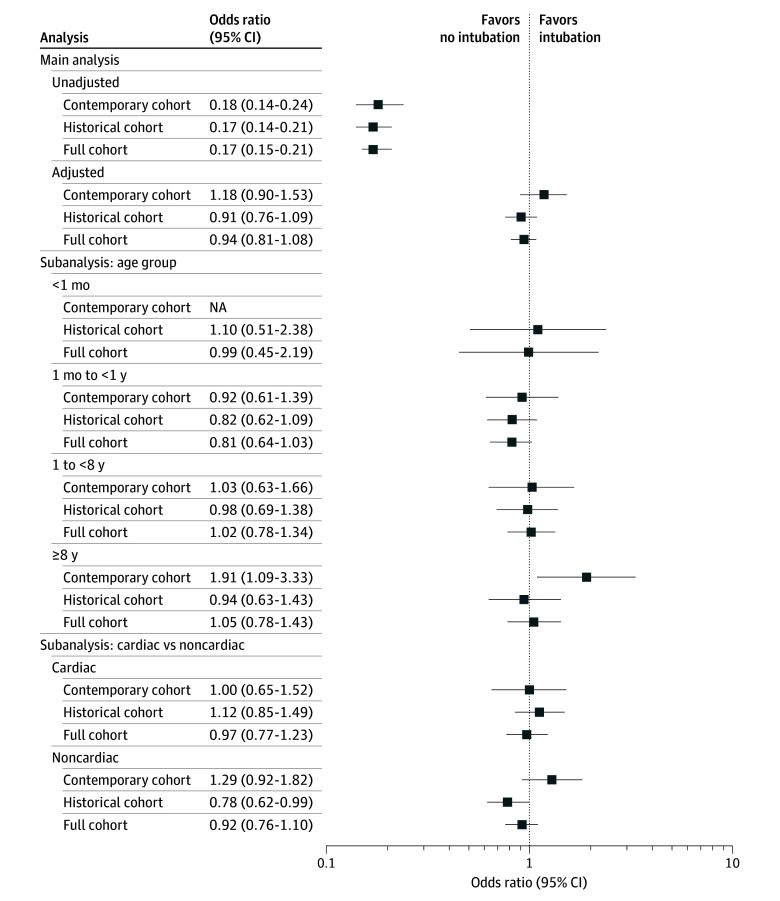
Intra-Arrest Intubation and Association With Survival to Hospital Discharge Unadjusted and adjusted analyses representing the association between intra-arrest intubation and survival to hospital discharge among the recent (2017-2022), historical (2000-2016), and full (2000-2022) cohorts.

In the unadjusted analysis, intra-arrest intubation compared with nonintubation in each minute was associated with decreased odds of survival to hospital discharge (odds ratio [OR], 0.18; 95% CI, 0.14-0.24; *P* < .001). The characteristics of the time-dependent propensity score matched cohort are shown in Table 2. The groups were well matched on all included variables. In the matched cohort, there was no association between intra-arrest intubation and survival to hospital discharge (adjusted OR [aOR], 1.18; 95% CI, 0.90-1.53; *P* = .23) (Figure 3) or ROSC (aOR, 1.05; 95% CI, 0.80-1.36; *P* = .74) (eFigure 2 in Supplement 1), with the point estimates in the direction favoring intubation.

**Table 2.** Characteristics of the Matched Cohort Restricted to the Recent (2017-2022) Cohort

Characteristic	Intubated (n = 500)	Not yet intubated (n = 500)	Standardized difference
Patient characteristics			
Age group			
<1 mo	1 (0.2)	1 (0.2)	0.000
1 mo to <1 y	207 (41.4)	207 (41.4)	0.000
1 to <8 y	154 (30.8)	154 (30.8)	0.000
≥8 y	138 (27.6)	138 (27.6)	0.000
Sex			
Female	230 (46.0)	244 (48.8)	−0.056
Male	270 (54.0)	256 (51.2)	0.056
Illness category			
Cardiac	197 (39.4)	197 (39.4)	0.000
Noncardiac	303 (60.6)	303 (60.6)	0.000
Preexisting conditions, No. (IQR)	2 (1-3)	2 (1-3)	0.004
Event characteristics			
Initial pulse status			
Pulseless	308 (61.6)	318 (63.6)	−0.041
Pulse (poor perfusion)	192 (38.4)	182 (36.4)	0.041
Initial rhythm			
Shockable	18 (3.6)	17 (3.4)	0.011
Nonshockable	440 (88.0)	442 (88.4)	−0.012
Unknown	42 (8.4)	41 (8.2)	0.007
Event witnessed	447 (89.4)	435 (87.0)	0.074
Location and time characteristics			
Event location			
Emergency department	134 (26.8)	144 (28.8)	−0.045
Intensive care unit	287 (57.4)	278 (55.6)	0.036
Floor (without telemetry)	53 (10.6)	50 (10.0)	0.020
Floor (with telemetry)	6 (1.2)	5 (1.0)	0.019
Other	20 (4.0)	23 (4.6)	−0.030
Weekday daytime (Monday through Friday, 7 am to 11 pm)	275 (55.0)	292 (58.4)	−0.069
Year			
2017	78 (15.6)	87 (17.4)	−0.049
2018	84 (16.8)	73 (14.6)	0.061
2019	100 (20.0)	90 (18.0)	0.051
2020	83 (16.6)	92 (18.4)	−0.047
2021	75 (15.0)	80 (16.0)	−0.028
2022	80 (16.0)	78 (15.6)	0.011
Hospital teaching status			
Major	279 (55.8)	285 (57.0)	−0.024
Minor	174 (34.8)	169 (33.8)	0.021
Nonteaching	5 (1.0)	4 (0.8)	0.021
Missing	42 (8.4)	42 (8.4)	0.000
Pediatric-only hospital	379 (75.8)	378 (75.6)	0.005

Subgroup analyses for survival to hospital discharge and ROSC are shown in Figures 3 and eFigure 2 in Supplement 1, respectively. In children 8 years or older, intubating compared to not intubating in a given minute was associated with increased odds of survival to hospital discharge (aOR, 1.91; 95% CI, 1.09-3.33; *P* = .02). In children with noncardiac illness category, there was no association between intra-arrest intubation and survival to hospital discharge (aOR, 1.29; 95% CI, 0.92-1.82; *P* = .14), with the point estimate in the direction favoring intubation.

The sensitivity analysis showing the association of intra-arrest intubation with survival with a favorable neurologic outcome, including the imputed missing neurologic outcome data, are shown in eFigure 3 in Supplement 1. Although there was no association between intra-arrest intubation and favorable neurologic outcome, the point estimate favored better outcomes in the recent cohort and worse outcomes in the historical cohort.

The sensitivity analysis computing the propensity score with competing risk analysis (ROSC and intubation treated as competing events) produced similar results to the cause-specific hazard model. Intra-arrest intubation was not associated with survival to hospital discharge (aOR, 1.10; 95% CI, 0.84-1.45; *P* = .48), except in children 8 years or older, for whom intubating compared with not intubating in each minute was associated with increased odds of survival to hospital discharge (aOR, 1.92; 95% CI, 1.07-3.47; *P* = .03).

The sensitivity analyses including time to epinephrine as a fixed effect in the mixed effects model produced similar results to the primary analysis. Intra-arrest intubation was not associated with survival to hospital discharge after controlling for early (≤2 minutes)^22^ epinephrine (aOR, 1.23; 95% CI, 0.91-1.66; *P* = .18) or late (>5 minutes)^13^ epinephrine (aOR, 1.29; 95% CI, 0.96-1.74; *P* = .10).

#### Historical Cohort (2000-2016)

In the unadjusted analysis, intubation compared to nonintubation in each minute was associated with decreased odds of survival to hospital discharge (OR, 0.17; 95% CI, 0.14-0.21; *P* < .001). The matched cohorts were well matched on all variables (eTable 2 in Supplement 1). In the matched cohort, there was no association between intra-arrest intubation and survival to hospital discharge (aOR, 0.91; 95% CI, 0.76-1.09; *P* = .30) (Figure 3) or ROSC (aOR, 1.02; 95% CI, 0.85-1.23; *P* = .79) (eFigure 3 in Supplement 1). In children with noncardiac illness category, intubation compared with delayed intubation was associated with decreased odds of survival to hospital discharge (aOR, 0.78; 95% CI, 0.62-0.99; *P* = .04).

#### Full Cohort (2000-2022)

In unadjusted analysis, intubating compared to not intubating in each minute was associated with decreased odds of survival to hospital discharge (OR 0.17, 95% CI: 0.15, 0.21, *P* < .001). The matched cohorts were well matched on all variables (eTable 3 in Supplement 1). In the matched cohort, there was no association between intra-arrest intubation and survival to hospital discharge (aOR 0.94, 95% CI: 0.81, 1.08, *P* = .38) (Figure 3) or ROSC (aOR 1.02, 95% CI: 0.88, 1.18, *P* = .78) (eFigure 3 in Supplement 1).

## Discussion

In this observational study of pediatric patients without an advanced airway at the start of an IHCA, we found that in a recent cohort (2017-2022), intra-arrest intubation rates have decreased and intubation occurs later during CPR compared with historical controls (2000-2016). In contrast to previous findings associating intra-arrest intubation with decreased risk of survival to hospital discharge,^8^ we did not find an association between intra-arrest intubation and survival to hospital discharge or ROSC for the patients in the recent cohort. In subgroup analysis, intubation compared with delayed intubation in children 8 years or older was associated with increased odds of survival to hospital discharge. These findings describe the landscape of pediatric IHCA intra-arrest intubation practice, reflect the current association between intra-arrest intubation and survival, and begin to identify specific subpopulations of patients that may benefit from intra-arrest intubation.

High-quality CPR is characterized by chest compressions with adequate rate, depth, recoil, and chest compression fraction (minimizing interruptions).^9^ Pauses in chest compressions, including for ventilation, can adversely affect hemodynamics.^23^ Importantly, pediatric intubation requires specialized skills and may require long pauses in chest compressions to successfully place an endotracheal tube during CPR.^24^ Additionally, skilled resources and attention to CPR quality may be diverted during an intubation attempt. Furthermore, there may be delayed recognition of complications of tracheal intubations, including esophageal intubation, given that pulmonary blood flow and therefore end-tidal carbon dioxide levels are lower during cardiac arrest. Although establishing an advanced airway for effective ventilation was previous dogma, we found decreasing rates of intra-arrest intubation and longer time to intubation during our study period. This may reflect the increased emphasis on CPR quality and thus may have mitigated some adverse effects of intra-arrest intubation on survival outcomes. Interestingly, although the intra-arrest intubation rate decreased overall during the study period, the intubation rate increased in the recent cohort. This trend will be important to evaluate in subsequent studies.

Whereas the only prior study of intra-arrest intubation in IHCA found a marginally significant association between intra-arrest intubation and decreased risk of survival to hospital discharge,^8^ we found no association in a recent cohort of patients enrolled in GWTG-R since that study. Some of the observed change in the association may be attributable to temporal trends in intra-arrest intubation and a more thoughtful or selective approach to intra-arrest intubation. Additionally, although both studies used time-dependent propensity score–matched analysis in order to account for resuscitation time bias,^25^ our study design explicitly excluded resuscitations related to birth that likely used neonatal (rather than pediatric) advanced life support algorithms. Consequently, more neonates younger than 1 month (with transitional newborn physiology and birth-related IHCAs) were excluded in our study, which may in part explain differences in conclusions. Furthermore, the random effects in the mixed effects model were able to account for the matched pairs and individual hospital sites, and we forced matching on specific characteristics in order to facilitate subgroup analyses, which may also partially explain differences in the association between intubation and outcomes.

There is substantial heterogeneity in terms of pediatric IHCA etiology and physiology, as well as among clinicians and settings in the approach to management and the available resources for emergent pediatric airway management. It is unlikely that a uniform approach would be appropriate for all patients or all IHCA care settings. Identifying specific subpopulations that may be helped or harmed by intra-arrest intubation may lead to personalized care and thus improved outcomes. Among children 8 years or older, intra-arrest intubation in a given minute compared with delaying intubation was associated with increased odds of survival to hospital discharge in the 2017-2022 cohort. Although this study is not designed to identify factors or physiologic mechanisms of survival benefit, prior studies of pediatric tracheal intubation for children without IHCA have identified associations between younger age and multiple intubation attempts, more severe desaturations, and more tracheal intubation-associated events.^26,27^ These findings suggest practical considerations that may at least partially explain why intra-arrest intubation may be harmful for some younger children. Additionally, clinicians frequently provide higher ventilation rates with an advanced airway than what guidelines recommend,^28,29^ which may have adverse hemodynamic effects.^30^ For noncardiac patients in the historical cohort, intra-arrest intubation compared to delayed intubation was associated with substantially worse odds of survival to hospital discharge. In contrast, that negative association was not evident in the recent cohort, suggesting potentially improved selectivity about whom to intubate or superior peri-intubation CPR quality and choreography to mitigate potential adverse effects. Further prospective work is necessary to better understand the physiologic mechanisms that may underlie the associations between intra-arrest intubation and survival outcomes in specific subpopulations of patients, including granular analysis of the number of intubation attempts, the duration of ventilation and CPR pauses for intubation, and changes in hemodynamics before and after intubation.

### Limitations

This study has some limitations. Importantly, the GWTG-R registry is a voluntary database. Participating institutions and nonparticipating institutions may differ, limiting generalizability. Furthermore, participating centers may not submit all IHCA events, introducing additional selection bias. Additionally, data collected during CPR is frequently not electronically timestamped, and thus the true accuracy of the resuscitation time data cannot be verified. Because this is an observational study, there are likely unmeasured confounders that cannot be controlled for, including most importantly the choice of who to intubate and why. Propensity score methods are constructed on observed variables, and unobserved confounders likely persist. The availability of variables for propensity score construction and matching are limited by what is available in the GWTG-R database. Additionally, the use of matching may exclude important observations. Other causal inference statistical methods were considered; however, time-dependent propensity score matching was chosen in order to contextualize our findings to the existing literature. Although a sensitivity analysis imputing best- and worst-case neurologic outcomes was intended to address missingness not at random (MNAR) for this particular variable, MNAR remains a potential factor for other variables in this observational dataset. Notably, we were unable to capture the number of intubation attempts, including unsuccessful intubations. Additionally, CPR quality metrics, duration of CPR pauses, and CPR physiology data were unavailable. Finally, this study may have been underpowered to detect clinically relevant associations, especially in the subgroup analyses, and thus risks failing to identify an association where one actually exists.

## Conclusion

In this cohort study of pediatric patients with IHCA, using a time-dependent propensity score matched analysis, we did not find an association between intra-arrest intubation and survival to hospital discharge. In subgroup analysis, intubation compared to delaying intubation in children aged 8 years or older was associated with increased odds of survival to hospital discharge. This study provides insight into temporal trends in pediatric intra-arrest intubation. Furthermore, we identified a subpopulation of older children who may benefit from intra-arrest intubation. Further investigation into the physiologic and practical mechanisms of this association is warranted.

## Notes

Supplement 1.
eMethods.
**eTable 1.** Characteristics of the full, recent, and historic study cohorts **eTable 2.** Characteristics of the matched cohort restricted to the historical (2000-2016) cohort **eTable 3.** Characteristics of the matched cohort in the full (2000-2022) cohort **eFigure 1.** Trends in intra-arrest advanced airway placement (less restrictive cohort) by year **eFigure 2.** Intra-arrest advanced airway placement and association with return of spontaneous circulation **eFigure 3.** Intra-arrest advanced airway placement and association with favorable neurologic outcome

Supplement 2.
Nonauthor Collaborators

Supplement 3.
Data Sharing Statement
