# Supplementary material for: Intubation Trends and Survival in Pediatric In-Hospital Cardiac Arrest
Source: JAMA Netw Open. 2025 Nov 20;8(11):e2544365. doi: 10.1001/jamanetworkopen.2025.44365 (PMC12635882; doi:10.1001/jamanetworkopen.2025.44365)
Supplement: Supplement 1. — eMethods. eTable 1. Characteristics of the full, recent, and historic study cohorts eTable 2. Characteristics of the matched cohort restricted to the historical (2000-2016) cohort eTable 3. Characteristics of the matched cohort in the full (2000-2022) cohort eFigure 1. Trends in intra-arrest advanced airway placement (less restrictive cohort) by year eFigure 2. Intra-arrest advanced airway placement and association with return of spontaneous circulation eFigure 3. Intra-arrest advanced airway placement and association with favorable neurologic outcome [file jamanetwopen-e2544365-s001.pdf]

Supplemental Online Content

Shepard LN, Reeder RW, Hsu J, et al. Intubation trends and survival in pediatric in-hospital cardiac arrest. *JAMA Netw Open*. 2025;8(11):e2544365. doi:10.1001/jamanetworkopen.2025.44365

eMethods.

eTable 1. Characteristics of the full, recent, and historic study cohorts

eTable 2. Characteristics of the matched cohort restricted to the historical (2000-2016) cohort

eTable 3. Characteristics of the matched cohort in the full (2000-2022) cohort

eFigure 1. Trends in intra-arrest advanced airway placement (less restrictive cohort) by year

eFigure 2. Intra-arrest advanced airway placement and association with return of spontaneous circulation

eFigure 3. Intra-arrest advanced airway placement and association with favorable neurologic outcome

This supplemental material has been provided by the authors to give readers additional information about their work.

© 2025 Shepard LN et al. JAMA Network Open.

Supplemental Material

eMethods

Annual trends in intra-arrest advanced airway placement were calculated by tabulating the rate of intra-arrest advanced airway placement for each year from 2000 to 2022 and assessed with Cochran-Armitage non-parametric trend test. Median time to intubation (minutes) by year was assessed with Jonckheere-Terpstra non-parametric trend test. A sensitivity analysis evaluated the trends in intra-arrest intubation including patients excluded from the primary analysis due to missing or inconsistent patient characteristics, intubation timing, or outcome data.

To evaluate the association between intra-arrest intubation and survival to hospital discharge, a time-dependent propensity score matched analysis was performed in the recent 2017-2022 cohort (after the Andersen et. al. publication),[1] as well as in a historic cohort (2000-2016) and the full cohort (2000-2022).[2] A propensity score for intra-arrest intubation was generated using a multivariable Cox regression model based on patient and pre-arrest clinical characteristics.[3] Variables included in the propensity score were selected *a priori* based on clinical reasoning and prior work;[1] these included age group,[4] sex, illness category (cardiac vs. non-cardiac),[5] number of pre-existing conditions, pulse status at the onset of CPR (pulseless vs. not pulseless),[6] initial rhythm (shockable vs. non-shockable),[7] event location, event time (weekday daytime vs. night or weekend),[8] event witnessed, hospital teaching status, pediatric-only hospital, and event year.[9] Patients intubated in each minute were matched with patients at risk of intubation (i.e., still receiving CPR and not yet intubated) at the same time point during the resuscitation. Matching was performed for each minute up to the 90th percentile of intra-arrest intubation timing in the cohort (19 minutes), as intubation beyond this time point became rare. Matching (1:1) was performed with forced matching on two *a priori* identified variables (age and illness categories) using nearest neighbor matching with a maximum caliper of 0.01 of the propensity score, allowing replacement of controls. Forced matching on age and cardiac vs. non-cardiac illness categories was designed to allow for subgroup analyses. Specifically, age was chosen given differences in pediatric anatomy by age,[10] and prior work showing an

© 2025 Shepard LN et al. JAMA Network Open.

association between age and number of intubation attempts, desaturation events, and tracheal intubation-associated events.[11,12] Cardiac vs. non-cardiac illness category was chosen based on differences in etiology of arrest and physiologic perturbations affecting cardiopulmonary interactions which necessitate special consideration in patients with cardiac disease.[5,13] To assess the performance of the match, standardized differences were calculated, with a threshold of <|0.1| considered negligible.[14] After matching, a mixed effects logistic regression model assessed the relationship between intra-arrest intubation and survival to hospital discharge, with weighting to account for the number of times a patient was included as a control. Whereas logistic regression or modified Poisson regression evaluates the average effect of the exposure in a population, mixed effects logistic regression was chosen to evaluate the effect of intubating in a given minute (as opposed to delaying intubation or not intubating altogether) on survival outcome for a given subject. Fixed effects were used to evaluate the effect of intra-arrest intubation on survival and to control for treatment at a high-volume center (defined as a median of ≥10 events per year of contribution to the database). Random effects were used to account for the matched data and clustering by hospital.

A pre-planned sensitivity analysis used competing risk analysis to generate the propensity score, treating ROSC as a competing risk for intra-arrest intubation.[15] Due to substantial missingness in the neurologic outcome variable (not at random), we performed a sensitivity analysis evaluating the association of intra-arrest intubation and survival with favorable neurologic outcome, with *post hoc* sensitivity analyses in which missing neurologic outcome was imputed as poor (worst case) and favorable (best case) for all missing subjects.

Given that time to epinephrine is associated with outcomes, two *post hoc* sensitivity analyses were performed including time to epinephrine as a fixed effect in the mixed effects model.[16,17] First, time to epinephrine was defined as early epinephrine (epinephrine given in ≤2 minutes).[16] Then, time to epinephrine was defined as late epinephrine (epinephrine given in >5 minutes).[17]

References

© 2025 Shepard LN et al. JAMA Network Open.

1. Andersen LW, Raymond TT, Berg RA, et al. Association Between Tracheal Intubation During Pediatric In-Hospital Cardiac Arrest and Survival. *JAMA*. Nov 1 2016;316(17):1786-1797. doi:10.1001/jama.2016.14486
2. Andersen LW, Grossestreuer AV, Donnino MW. "Resuscitation time bias"-A unique challenge for observational cardiac arrest research. *Resuscitation*. Apr 2018;125:79-82. doi:10.1016/j.resuscitation.2018.02.006
3. Lu B. Propensity score matching with time-dependent covariates. *Biometrics*. Sep 2005;61(3):721-8. doi:10.1111/j.1541-0420.2005.00356.x
4. Meaney PA, Nadkarni VM, Cook EF, et al. Higher survival rates among younger patients after pediatric intensive care unit cardiac arrests. *Pediatrics*. Dec 2006;118(6):2424-33. doi:10.1542/peds.2006-1724
5. Matos RI, Watson RS, Nadkarni VM, et al. Duration of cardiopulmonary resuscitation and illness category impact survival and neurologic outcomes for in-hospital pediatric cardiac arrests. *Circulation*. Jan 29 2013;127(4):442-51. doi:10.1161/CIRCULATIONAHA.112.125625
6. Morgan RW, Reeder RW, Meert KL, et al. Survival and Hemodynamics During Pediatric Cardiopulmonary Resuscitation for Bradycardia and Poor Perfusion Versus Pulseless Cardiac Arrest. *Crit Care Med*. Jun 2020;48(6):881-889. doi:10.1097/CCM.0000000000004308
7. Nadkarni VM, Larkin GL, Peberdy MA, et al. First documented rhythm and clinical outcome from in-hospital cardiac arrest among children and adults. *JAMA*. Jan 4 2006;295(1):50-7. doi:10.1001/jama.295.1.50
8. Bhanji F, Topjian AA, Nadkarni VM, et al. Survival Rates Following Pediatric In-Hospital Cardiac Arrests During Nights and Weekends. *JAMA Pediatr*. Jan 1 2017;171(1):39-45. doi:10.1001/jamapediatrics.2016.2535
9. Girotra S, Spertus JA, Li Y, et al. Survival trends in pediatric in-hospital cardiac arrests: an analysis from Get With the Guidelines-Resuscitation. *Circ Cardiovasc Qual Outcomes*. Jan 1 2013;6(1):42-9. doi:10.1161/CIRCOUTCOMES.112.967968
10. Adewale L. Anatomy and assessment of the pediatric airway. *Paediatr Anaesth*. Jul 2009;19 Suppl 1:1-8. doi:10.1111/j.1460-9592.2009.03012.x
11. Lee JH, Turner DA, Kamat P, et al. The number of tracheal intubation attempts matters! A prospective multi-institutional pediatric observational study. *BMC Pediatr*. Apr 29 2016;16:58. doi:10.1186/s12887-016-0593-y
12. Parker MM, Nuthall G, Brown C, 3rd, et al. Relationship Between Adverse Tracheal Intubation Associated Events and PICU Outcomes. *Pediatr Crit Care Med*. Apr 2017;18(4):310-318. doi:10.1097/PCC.0000000000001074
13. Marino BS, Tabbutt S, MacLaren G, et al. Cardiopulmonary Resuscitation in Infants and Children With Cardiac Disease: A Scientific Statement From the American Heart Association. *Circulation*. May 29 2018;137(22):e691-e782. doi:10.1161/CIR.0000000000000524
14. Haukoos JS, Lewis RJ. The Propensity Score. *JAMA*. Oct 20 2015;314(15):1637-8. doi:10.1001/jama.2015.13480
15. Fine JP, Gray RJ. A proportional hazards model for the subdistribution of a competing risk. *Journal of the Americal Statistical Association*. 1999 1999;94:496-509.
16. Siems A, Naim MY, Berg RA, et al. Association of Early Epinephrine with Hemodynamics and Outcome in Pediatric In-Hospital Cardiac Arrest: A Secondary Analysis of a Multi-center, Cluster-randomized Clinical Trial (ICU-RESUS). *Ann Am Thorac Soc*. Jun 4 2025;doi:10.1513/AnnalsATS.202408-825OC
17. Andersen LW, Berg KM, Saindon BZ, et al. Time to Epinephrine and Survival After Pediatric In-Hospital Cardiac Arrest. *JAMA*. Aug 25 2015;314(8):802-10. doi:10.1001/jama.2015.9678

© 2025 Shepard LN et al. JAMA Network Open.

© 2025 Shepard LN et al. JAMA Network Open.

eTable 1. Characteristics of the full, recent, and historic study cohorts.

		Full Cohort (2000-2022)		Recent Cohort (2017-2022)		Historic Cohort (2000-2016)	
		Intubated	Not Intubated	Intubated	Not Intubated	Intubated	Not Intubated
		N=2,164	N=1,098	N=718	N=474	N=1,446	N=624
Patient Characteristics							
Age (months [IQR])		13.0 [4.0, 95.8]	9.0 [3.0, 59.9]	19.5 [6.0, 107.8]	9.5 [3.0, 19.5]	13.0 [3.0, 95.8]	9.0 [3.0, 59.9]
Age Group	<1 month	118 (5.5%)	62 (5.6%)	5 (0.7%)	12 (2.5%)	113 (7.8%)	50 (8.0%)
	1 month to <1 year	813 (37.6%)	515 (46.9%)	279 (38.9%)	232 (48.9%)	534 (36.9%)	283 (45.4%)
	1 to <8 years	658 (30.4%)	288 (26.2%)	231 (32.2%)	126 (26.6%)	427 (29.5%)	162 (26.0%)
	>=8 years	575 (26.6%)	233 (21.2%)	203 (28.3%)	104 (21.9%)	372 (25.7%)	129 (20.7%)
Female		982 (45.4%)	505 (46.0%)	333 (46.4%)	218 (46.0%)	649 (44.9%)	287 (46.0%)
Illness Category	Cardiac	913 (42.2%)	390 (35.5%)	300 (41.8%)	171 (36.1%)	613 (42.4%)	219 (35.1%)
	Non-cardiac	1,251 (57.8%)	708 (64.5%)	418 (58.2%)	303 (63.9%)	833 (57.6%)	405 (64.9%)
Number of Pre-existing Conditions (N [IQR])		1 [1, 3]	2 [1, 2]	2 [1, 3]	2 [1, 3]	1 [1, 2]	1 [1, 2]
	None	266 (13.0%)	128 (11.9%)	89 (12.4%)	60 (12.7%)	177 (13.2%)	68 (11.2%)
	Respiratory Disorder[a]	1,033 (50.3%)	565 (52.4%)	372 (52.0%)	252 (53.2%)	661 (49.4%)	313 (51.7%)
	Cardiac Disorder[b]	737 (35.9%)	370 (34.3%)	255 (35.6%)	179 (37.8%)	482 (36.0%)	191 (31.6%)
	Neurologic Disorder[c]	449 (21.9%)	238 (22.1%)	176 (24.6%)	120 (25.3%)	273 (20.4%)	118 (19.5%)
	Shock	364 (17.7%)	152 (14.1%)	137 (19.1%)	67 (14.1%)	227 (17.0%)	85 (14.0%)
	Congenital Disorder[d]	258 (12.6%)	224 (20.8%)	140 (19.6%)	126 (26.6%)	118 (8.8%)	98 (16.2%)
	Endocrine/Metabolic/ Electrolyte Disorder[e]	318 (15.5%)	145 (13.4%)	147 (20.5%)	87 (18.4%)	171 (12.8%)	58 (9.6%)
	Sepsis	213 (10.4%)	89 (8.2%)	65 (9.1%)	34 (7.2%)	148 (11.1%)	55 (9.1%)
	Renal Disorder[f]	175 (8.5%)	72 (6.7%)	63 (8.8%)	32 (6.8%)	112 (8.4%)	40 (6.6%)
	Oncologic Disorder[g]	129 (6.3%)	57 (5.3%)	47 (6.6%)	27 (5.7%)	82 (6.1%)	30 (5.0%)
	Hepatic Disorder[h]	89 (4.3%)	31 (2.9%)	41 (5.7%)	15 (3.2%)	48 (3.6%)	16 (2.6%)
	Missing Pre-existing Conditions	110 (5.1%)	19 (1.7%)	2 (0.3%)	0 (0.0%)	108 (7.5%)	19 (3.0%)
Event Characteristics							
Initial Pulse Status	Pulseless	1,261 (58.3%)	504 (45.9%)	459 (63.9%)	244 (51.5%)	802 (55.5%)	260 (41.7%)
	Pulse (Poor Perfusion)	903 (41.7%)	594 (54.1%)	259 (36.1%)	230 (48.5%)	644 (44.5%)	364 (58.3%)
Initial Rhythm	Shockable	127 (5.9%)	64 (5.8%)	36 (5.0%)	25 (5.3%)	91 (6.3%)	39 (6.2%)
	Non-shockable	1,838 (84.9%)	895 (81.5%)	617 (85.9%)	394 (83.1%)	1,221 (84.4%)	501 (80.3%)
	Unknown	199 (9.2%)	139 (12.7%)	65 (9.1%)	55 (11.6%)	134 (9.3%)	84 (13.5%)
Event Witnessed		1,908 (88.2%)	1,000 (91.1%)	636 (88.6%)	430 (90.7%)	1,272 (88.0%)	570 (91.3%)
Location and Time Characteristics							
Event Location	Emergency Department	522 (24.1%)	118 (10.7%)	196 (27.3%)	54 (11.4%)	326 (22.5%)	64 (10.3%)

© 2025 Shepard LN et al. JAMA Network Open.

	Intensive Care Unit	944 (43.6%)	553 (50.4%)	392 (54.6%)	270 (57.0%)	552 (38.2%)	283 (45.4%)
	Floor (without telemetry)	288 (13.3%)	220 (20.0%)	89 (12.4%)	96 (20.3%)	199 (13.8%)	124 (19.9%)
	Floor (with telemetry)	82 (3.8%)	65 (5.9%)	12 (1.7%)	24 (5.1%)	70 (4.8%)	41 (6.6%)
	Other	328 (15.2%)	142 (12.9%)	29 (4.0%)	30 (6.3%)	299 (20.7%)	112 (17.9%)
Weekday Daytime (Mon-Fri, 7am to 11pm)		1,160 (53.6%)	576 (52.5%)	397 (55.3%)	250 (52.7%)	763 (52.8%)	326 (52.2%)
Year	2000	33 (1.5%)	6 (0.5%)			33 (2.3%)	6 (1.0%)
	2001	46 (2.1%)	9 (0.8%)			46 (3.2%)	9 (1.4%)
	2002	59 (2.7%)	9 (0.8%)			59 (4.1%)	9 (1.4%)
	2003	66 (3.0%)	22 (2.0%)			66 (4.6%)	22 (3.5%)
	2004	97 (4.5%)	15 (1.4%)			97 (6.7%)	15 (2.4%)
	2005	87 (4.0%)	39 (3.6%)			87 (6.0%)	39 (6.2%)
	2006	89 (4.1%)	32 (2.9%)			89 (6.2%)	32 (5.1%)
	2007	101 (4.7%)	34 (3.1%)			101 (7.0%)	34 (5.4%)
	2008	127 (5.9%)	45 (4.1%)			127 (8.8%)	45 (7.2%)
	2009	125 (5.8%)	36 (3.3%)			125 (8.6%)	36 (5.8%)
	2010	94 (4.3%)	43 (3.9%)			94 (6.5%)	43 (6.9%)
	2011	75 (3.5%)	36 (3.3%)			75 (5.2%)	36 (5.8%)
	2012	84 (3.9%)	40 (3.6%)			84 (5.8%)	40 (6.4%)
	2013	82 (3.8%)	41 (3.7%)			82 (5.7%)	41 (6.6%)
	2014	90 (4.2%)	64 (5.8%)			90 (6.2%)	64 (10.3%)
	2015	101 (4.7%)	67 (6.1%)			101 (7.0%)	67 (10.7%)
	2016	90 (4.2%)	86 (7.8%)			90 (6.2%)	86 (13.8%)
	2017	116 (5.4%)	91 (8.3%)	116 (16.2%)	91 (19.2%)		
	2018	117 (5.4%)	105 (9.6%)	117 (16.3%)	105 (22.2%)		
	2019	135 (6.2%)	76 (6.9%)	135 (18.8%)	76 (16.0%)		
	2020	120 (5.5%)	79 (7.2%)	120 (16.7%)	79 (16.7%)		
	2021	118 (5.5%)	67 (6.1%)	118 (16.4%)	67 (14.1%)		
	2022	112 (5.2%)	56 (5.1%)	112 (15.6%)	56 (11.8%)		
Hospital Teaching Status	Major	1,070 (49.7%)	641 (58.8%)	381 (53.1%)	289 (61.0%)	689 (47.9%)	352 (57.1%)
	Minor	718 (33.3%)	324 (29.7%)	246 (34.3%)	139 (29.3%)	472 (32.8%)	185 (30.0%)
	Non-teaching	66 (3.1%)	10 (0.9%)	26 (3.6%)	4 (0.8%)	40 (2.8%)	6 (1.0%)
	Missing	301 (14.0%)	115 (10.6%)	65 (9.1%)	42 (8.9%)	236 (16.4%)	73 (11.9%)
Pediatric Only Hospital		1,598 (74.2%)	857 (78.6%)	551 (76.7%)	368 (77.6%)	1,047 (72.9%)	489 (79.4%)
Event Outcome							
Event Duration (min [IQR])		28 [13, 47]	3 [2, 7]	30 [14, 50]	3 [2, 7]	26 [13, 45]	3 [2, 8]
Immediate Outcome	ROSC	1,215 (56.1%)	1,011 (92.1%)	384 (53.5%)	439 (92.6%)	831 (57.5%)	572 (91.7%)
	ROC via ECPR	223 (10.3%)	7 (0.6%)	112 (15.6%)	4 (0.8%)	111 (7.7%)	3 (0.5%)

© 2025 Shepard LN et al. JAMA Network Open.

	Died	726 (33.5%)	80 (7.3%)	222 (30.9%)	31 (6.5%)	504 (34.9%)	49 (7.9%)
Survival to Hospital Discharge		874 (40.4%)	874 (79.6%)	306 (42.6%)	380 (80.2%)	568 (39.3%)	494 (79.2%)

ROSC = sustained return of spontaneous circulation; ECPR = extracorporeal cardiopulmonary resuscitation

[a] Pre-event respiratory insufficiency or pneumonia

[b] Pre-event cyanotic or acyanotic cardiac malformation, heart failure, or myocardial infarction

[c] Pre-event acute stroke or non-stroke neurologic event or baseline depression in neurologic function

[d] Non-cardiac congenital malformation

[e] Pre-event metabolic or electrolyte abnormality or diabetes mellitus

[f] Pre-event renal insufficiency

[g] Pre-event metastatic or hematologic malignancy

[h] Pre-event hepatic insufficiency

© 2025 Shepard LN et al. JAMA Network Open.

eTable 2. Characteristics of the matched cohort restricted to the historical (2000-2016) cohort.

		Intubated<br>N=1,113	Not Yet Intubated<br>N=1,113	Standardized Difference
Patient Characteristics				
Age Group	<1 month	59 (5.3%)	59 (5.3%)	0.000
	1 month to <1 year	440 (39.5%)	440 (39.5%)	0.000
	1 to <8 years	330 (29.6%)	330 (29.6%)	0.000
	≥8 years	284 (25.5%)	284 (25.5%)	0.000
Female		510 (45.8%)	479 (43.0%)	0.056
Illness Category	Cardiac	457 (41.1%)	457 (41.1%)	0.000
	Non-cardiac	656 (58.9%)	656 (58.9%)	0.000
Number of Pre-existing Conditions (N [IQR])		1 [1, 2]	1 [1, 2]	0.023
Event Characteristics				
Initial Pulse Status	Pulseless	606 (54.4%)	633 (56.9%)	-0.049
	Pulse (Poor Perfusion)	507 (45.6%)	480 (43.1%)	0.049
Initial Rhythm	Shockable	67 (6.0%)	62 (5.6%)	0.019
	Non-shockable	953 (85.6%)	957 (86.0%)	-0.010
	Unknown	93 (8.4%)	94 (8.4%)	-0.003
Event Witnessed		995 (89.4%)	968 (87.0%)	0.075
Location and Time Characteristics				
Event Location	Emergency Department	223 (20.0%)	211 (19.0%)	0.027
	Intensive Care Unit	460 (41.3%)	439 (39.4%)	0.038
	Floor (without telemetry)	154 (13.8%)	174 (15.6%)	-0.051
	Floor (with telemetry)	43 (3.9%)	51 (4.6%)	-0.036
	Other	233 (20.9%)	238 (21.4%)	-0.011
Weekday Daytime (Mon-Fri, 7am to 11pm)		597 (53.6%)	593 (53.3%)	0.007
Year	2000	25 (2.2%)	28 (2.5%)	-0.018
	2001	35 (3.1%)	46 (4.1%)	-0.053
	2002	43 (3.9%)	51 (4.6%)	-0.036
	2003	49 (4.4%)	49 (4.4%)	0.000
	2004	53 (4.8%)	59 (5.3%)	-0.025
	2005	71 (6.4%)	71 (6.4%)	0.000

© 2025 Shepard LN et al. JAMA Network Open.

	2006	70 (6.3%)	59 (5.3%)	0.042
	2007	69 (6.2%)	68 (6.1%)	0.004
	2008	96 (8.6%)	106 (9.5%)	-0.031
	2009	92 (8.3%)	93 (8.4%)	-0.003
	2010	70 (6.3%)	71 (6.4%)	-0.004
	2011	60 (5.4%)	58 (5.2%)	0.008
	2012	71 (6.4%)	66 (5.9%)	0.019
	2013	71 (6.4%)	58 (5.2%)	0.050
	2014	72 (6.5%)	68 (6.1%)	0.015
	2015	89 (8.0%)	86 (7.7%)	0.010
	2016	77 (6.9%)	76 (6.8%)	0.004
Hospital Teaching Status	Major	553 (49.7%)	530 (47.6%)	0.041
	Minor	363 (32.6%)	357 (32.1%)	0.012
	Non-teaching	18 (1.6%)	23 (2.1%)	-0.033
	Missing	179 (16.1%)	203 (18.2%)	-0.057
Pediatric-only Hospital		820 (73.7%)	800 (71.9%)	0.040

© 2025 Shepard LN et al. JAMA Network Open.

eTable 3. Characteristics of the matched cohort in the full (2000-2022) cohort.

		Intubated<br>N=1,747	Not Yet Intubated<br>N=1,747	Standardized Difference
Patient Characteristics				
Age Group	<1 month	59 (3.4%)	59 (3.4%)	0.000
	1 month to <1 year	690 (39.5%)	690 (39.5%)	0.000
	1 to <8 years	539 (30.9%)	539 (30.9%)	0.000
	≥8 years	459 (26.3%)	459 (26.3%)	0.000
Female		788 (45.1%)	844 (48.3%)	-0.064
Illness Category	Cardiac	725 (41.5%)	725 (41.5%)	0.000
	Non-cardiac	1,022 (58.5%)	1,022 (58.5%)	0.000
Number of Pre-existing Conditions (N [IQR])		1 [1, 2]	1 [1, 2]	0.038
Event Characteristics				
Initial Pulse Status	Pulseless	1,012 (57.9%)	987 (56.5%)	0.029
	Pulse (Poor Perfusion)	735 (42.1%)	760 (43.5%)	-0.029
Initial Rhythm	Shockable	101 (5.8%)	90 (5.2%)	0.028
	Non-shockable	1,495 (85.6%)	1,515 (86.7%)	-0.033
	Unknown	151 (8.6%)	142 (8.1%)	0.019
Event Witnessed		1,546 (88.5%)	1,555 (89.0%)	-0.016
Location and Time Characteristics				
Event Location	Emergency Department	401 (23.0%)	396 (22.7%)	0.007
	Intensive Care Unit	802 (45.9%)	825 (47.2%)	-0.026
	Floor (without telemetry)	230 (13.2%)	232 (13.3%)	-0.003
	Floor (with telemetry)	63 (3.6%)	65 (3.7%)	-0.006
	Other	251 (14.4%)	229 (13.1%)	0.037
Weekday Daytime (Mon-Fri, 7am to 11pm)		937 (53.6%)	949 (54.3%)	-0.014
Year	2000	28 (1.6%)	23 (1.3%)	0.024
	2001	38 (2.2%)	24 (1.4%)	0.061
	2002	45 (2.6%)	38 (2.2%)	0.026
	2003	46 (2.6%)	54 (3.1%)	-0.027
	2004	47 (2.7%)	48 (2.7%)	-0.004
	2005	72 (4.1%)	59 (3.4%)	0.039

© 2025 Shepard LN et al. JAMA Network Open.

	2006	69 (3.9%)	59 (3.4%)	0.030
	2007	73 (4.2%)	77 (4.4%)	-0.011
	2008	103 (5.9%)	110 (6.3%)	-0.017
	2009	96 (5.5%)	103 (5.9%)	-0.017
	2010	77 (4.4%)	83 (4.8%)	-0.016
	2011	62 (3.5%)	71 (4.1%)	-0.027
	2012	72 (4.1%)	70 (4.0%)	0.006
	2013	72 (4.1%)	74 (4.2%)	-0.006
	2014	75 (4.3%)	81 (4.6%)	-0.017
	2015	91 (5.2%)	84 (4.8%)	0.018
	2016	81 (4.6%)	77 (4.4%)	0.011
	2017	104 (6.0%)	108 (6.2%)	-0.010
	2018	99 (5.7%)	104 (6.0%)	-0.012
	2019	116 (6.6%)	111 (6.4%)	0.012
	2020	101 (5.8%)	108 (6.2%)	-0.017
	2021	90 (5.2%)	89 (5.1%)	0.003
	2022	90 (5.2%)	92 (5.3%)	-0.005
Hospital Teaching Status	Major	887 (50.8%)	880 (50.4%)	0.008
	Minor	579 (33.1%)	569 (32.6%)	0.012
	Non-teaching	33 (1.9%)	32 (1.8%)	0.004
	Missing	248 (14.2%)	266 (15.2%)	-0.029
Pediatric-only Hospital		1,304 (74.6%)	1,310 (75.0%)	-0.008

© 2025 Shepard LN et al. JAMA Network Open.

eFigure 1. Trends in intra-arrest advanced airway placement (less restrictive cohort) by year. Trends in intra-arrest intubation by year in the primary cohort (the final cohort included in the study) are shown with the light blue closed circles. A sensitivity analysis using a less restrictive cohort (i.e., including patients excluded from the primary analysis due to missing or inconsistent data) are shown in the dark blue open circles, showing that trends were similar to the final primary analytic cohort.

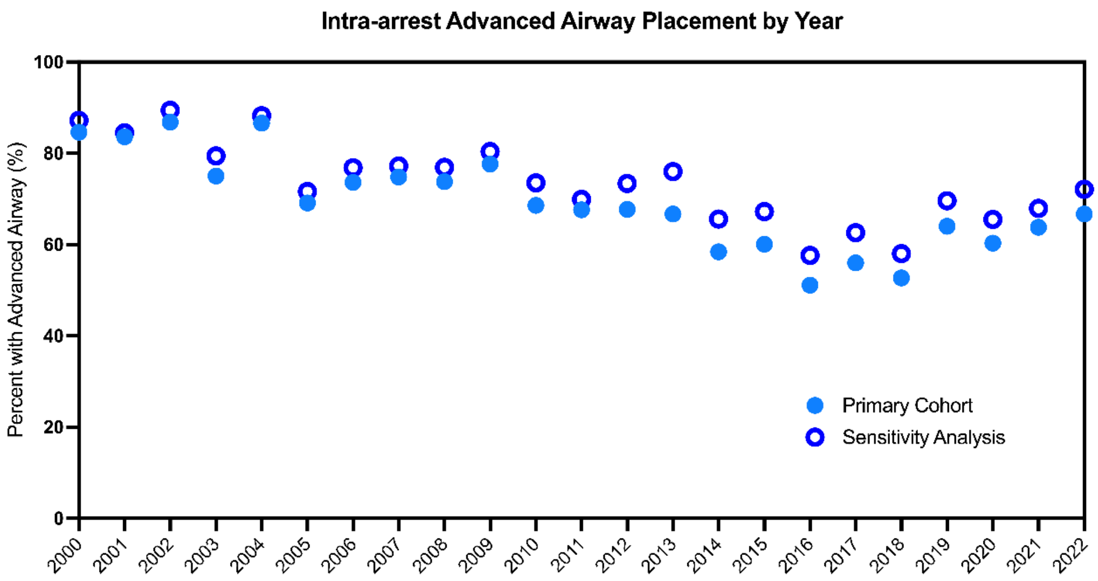

Primary Cohort

Year	2000	2001	2002	2003	2004	2005	2006	2007	2008	2009	2010	2011	2012	2013	2014	2015	2016	2017	2018	2019	2020	2021	2022
N	39	55	68	88	112	126	121	135	172	161	137	111	124	123	154	168	176	207	222	211	199	185	168
Percent with Advanced Airway (%)	84.6	83.6	86.8	75.0	86.6	69.1	73.6	74.8	73.8	77.6	68.6	67.6	67.7	66.7	58.4	60.1	51.1	56.0	52.7	64.0	60.3	63.8	66.7

Sensitivity Analysis

Year	2000	2001	2002	2003	2004	2005	2006	2007	2008	2009	2010	2011	2012	2013	2014	2015	2016	2017	2018	2019	2020	2021	2022
Number of Events	47	58	85	107	127	141	138	149	195	184	162	123	158	175	186	204	203	246	250	250	229	212	201
Percent with Advanced Airway (%)	87.2	84.5	89.4	79.4	88.2	71.6	76.8	77.2	76.9	80.4	73.5	69.9	73.4	76.0	65.6	67.2	57.6	62.6	58.0	69.6	65.5	67.9	72.1

© 2025 Shepard LN et al. JAMA Network Open.

eFigure 2. Intra-arrest advanced airway placement and association with return of spontaneous circulation.

Unadjusted and adjusted analyses of overall cohort as well as subgroup adjusted analyses by age group and illness category. The light blue dots show the unadjusted and adjusted odds ratios (and 95% confidence intervals) representing the association between intra-arrest intubation and return of spontaneous circulation among the recent cohort (2017-2022). The dark blue triangles show the association between intra-arrest intubation and return of spontaneous circulation among the historical cohort (2000-2016), and the gray squares show the association between intra-arrest intubation and return of spontaneous circulation among the full cohort (2000-2022).

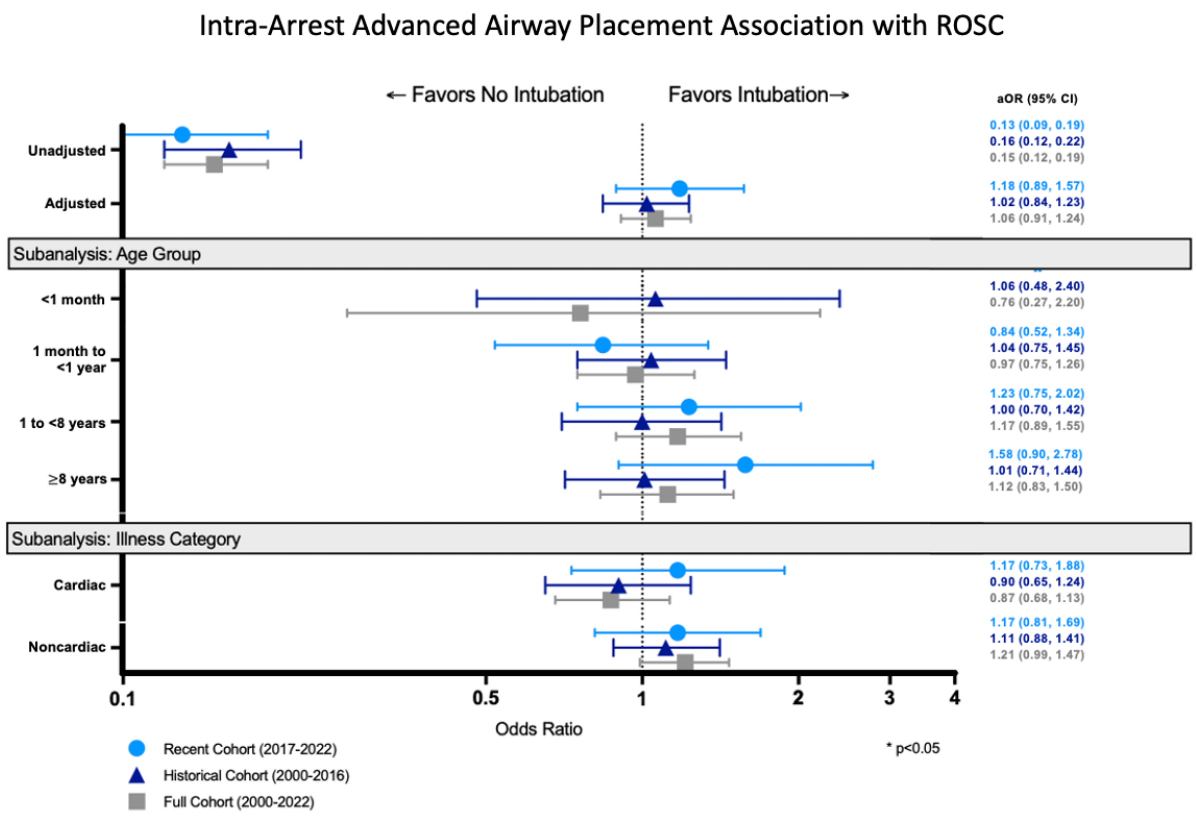

© 2025 Shepard LN et al. JAMA Network Open.

eFigure 3. Intra-arrest advanced airway placement and association with favorable neurologic outcome. The adjusted odds ratio (with 95% confidence interval) showing the association between intra-arrest intubation and favorable neurologic outcome is shown by the black circles. Given the substantial missingness in the favorable neurologic outcome variable, the best and worst case scenarios were imputed. The light gray triangles show the best case scenario, with all missing values imputed as favorable neurologic outcome. The dark black squares show the worst case scenario, with all missing values imputed as poor neurologic outcome. There was no statistically significant association between intra-arrest intubation and favorable neurologic outcome in the recent (2017-2022), historical (2000-2016) or full (2000-2022) cohorts, including after imputing missing values.

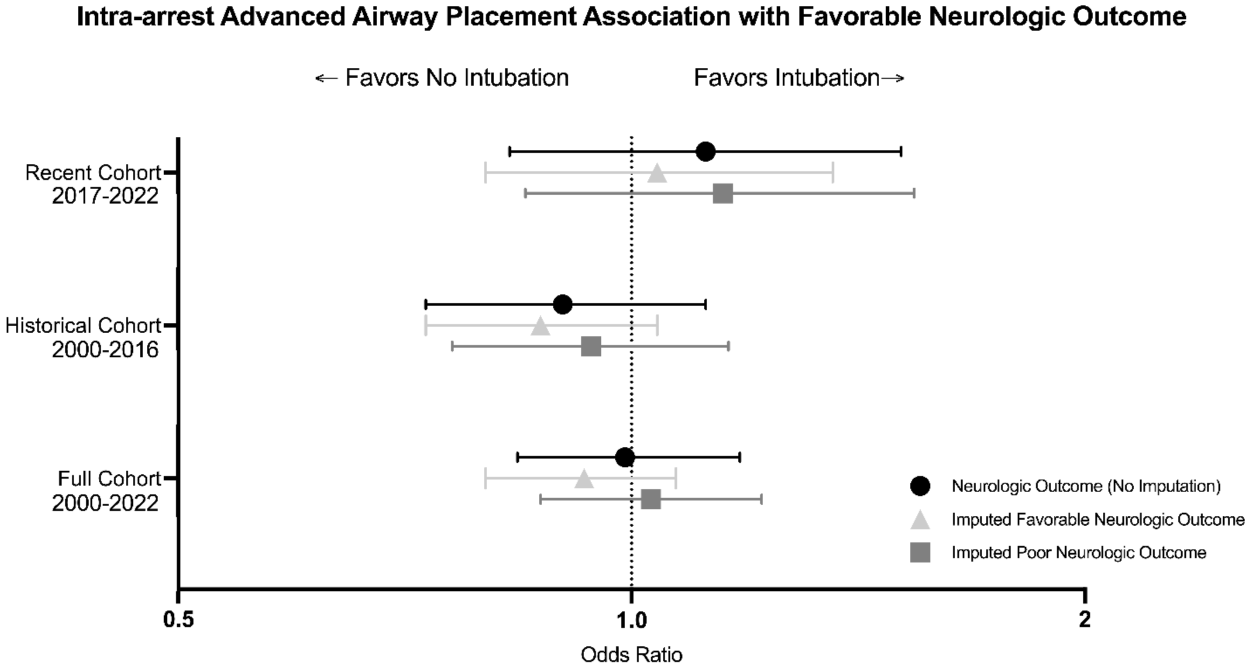

© 2025 Shepard LN et al. JAMA Network Open.
