# Supplementary material for: Intubation Trends and Survival in Pediatric In-Hospital Cardiac Arrest
Source: JAMA Netw Open. 2025 Nov 20;8(11):e2544365. doi: 10.1001/jamanetworkopen.2025.44365 (PMC12635882; doi:10.1001/jamanetworkopen.2025.44365)
Supplement: Supplement 2. — Nonauthor Collaborators [file jamanetwopen-e2544365-s002.pdf]

Supplemental Online Content: Nonauthor Collaborators
*First name, last name, and suffix (if applicable) are required and will appear in PubMed.

*Group Name(s): American Heart Association's Get With The Guidelines–Resuscitation Investigators							
***First Name and Middle Initial(s)**	***Last Name**	***Suffix (eg, Jr, III)**	Academic Degrees	Institution	Location (city, state/province, country)	Role or Contribution, eg, chair, principal investigator	Group (if more than 1 Group listed in the byline) and/or Subgroup (eg, Steering Committee)
Anne-Marie	Guerguerian		MD PhD FRCPC FAAP FAHA				
Caitlin E.	O'Brien		MD MPH				
Ericka L.	Fink		MD MS				
Javier J.	Lasa		MD FAAP				
Joan S.	Roberts		MD				
Lillian	Su		MD				
Linda L.	Brown		MD MSCE				
Maya	Dewan		MD MPH				
Monica	Kleinman		MD				
Noorjahan	Ali		MD MS FAAP				
Punkaj	Gupta		MBBS				
Robert M.	Sutton		MD MSCE				
Ron	Reeder		MS PhD				
Todd	Sweberg		MD MBA				
