# Supplementary material for: Intubation Trends and Survival in Pediatric In-Hospital Cardiac Arrest
Source: JAMA Netw Open. 2025 Nov 20;8(11):e2544365. doi: 10.1001/jamanetworkopen.2025.44365 (PMC12635882; doi:10.1001/jamanetworkopen.2025.44365)
Supplement: Supplement 3. — Data Sharing Statement [file jamanetwopen-e2544365-s003.pdf]

Data Sharing Statement

Shepard. Intubation Trends and Survival in Pediatric In-Hospital Cardiac Arrest. *JAMA Netw Open*. Published November 20, 2025. doi:10.1001/jamanetworkopen.2025.44365

Data
Data available: No

Additional Information
Explanation for why data not available: The data is owned by the American Heart Association, thus cannot be made available to others without written permission.
